# Making Mitotic Chromosomes in a Test Tube

**DOI:** 10.3390/epigenomes6030020

**Published:** 2022-07-20

**Authors:** Keishi Shintomi

**Affiliations:** Chromosome Dynamics Laboratory, RIKEN, Wako 351-0198, Saitama, Japan; kshintomi@riken.jp

**Keywords:** mitotic chromosome, *Xenopus* egg extract, reconstitution, condensin, topoisomerase II, histone

## Abstract

Mitotic chromosome assembly is an essential preparatory step for accurate transmission of the genome during cell division. During the past decades, biochemical approaches have uncovered the molecular basis of mitotic chromosomes. For example, by using cell-free assays of frog egg extracts, the condensin I complex central for the chromosome assembly process was first identified, and its functions have been intensively studied. A list of chromosome-associated proteins has been almost completed, and it is now possible to reconstitute structures resembling mitotic chromosomes with a limited number of purified factors. In this review, I introduce how far we have come in understanding the mechanism of chromosome assembly using cell-free assays and reconstitution assays, and I discuss their potential applications to solve open questions.

## 1. Introduction

When cells enter mitosis, chromatin fibers begin to condense within the nucleus, transforming into thread-like structures. Later, as the nuclear envelope breaks down, the thread-like structures become thicker and shorter, resulting in highly organized structures known as mitotic chromosomes. This process, called mitotic chromosome assembly or chromosome condensation, is essential for the accurate transmission of sister chromatids to daughter cells. Numerous efforts have been made over the past century to elucidate the mechanisms underlying this process. Model organisms and experimental approaches used in chromosome biology are diverse. For example, mutant screening by yeast genetics and microscopic analyses in mammalian tissue culture cells have yielded many important findings. However, we should not forget that biochemical approaches have also made substantial contributions. The condensin complex, which plays a central role in mitotic chromosome assembly, was discovered by a proteomic analysis of chromosomes assembled in a cell-free extract of frog eggs [1,2]. More recently, using only purified proteins, we have succeeded in reconstituting structures nearly equivalent to chromosomes assembled in the cell-free assays [3]. In this review, first, the outline of the frog egg extracts and the reconstitution assays as well as their historical background are introduced. Second, with emphasis on these highly tractable in vitro assays, the progress in mitotic chromosome research is summarized. Lastly, possible strategies to solve the remaining mysteries are discussed.

## 2. Establishment of Frog Egg Extracts for Studying Large-Scale Chromatin Structures

**Figure 1:**
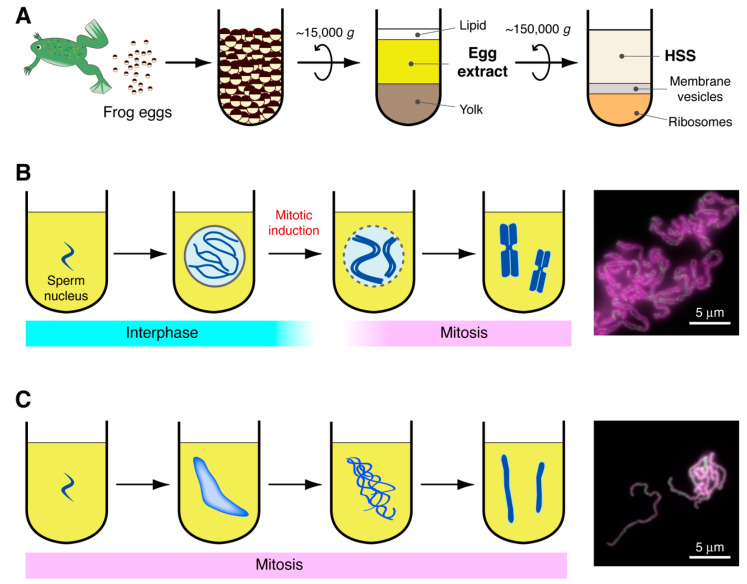
Cell-free chromosome assembly assays using frog egg extracts. (**A**) Preparation of egg extracts. The cytoplasmic fraction recovered after low-speed centrifugation can be directly used as an egg extract for both duplicated chromosome assembly and single chromatid assembly and assays. The high-speed supernatant (HSS) of an egg extract supports only single chromatid assembly. (**B**) The duplicated chromosome assembly assay. The resultant structures were fixed and labeled by anti-CAP-G antibody (green) and DAPI (magenta). (**C**) The single chromatid assembly assay. The resultant structures were processed as in (**B**).

**Figure 2:**
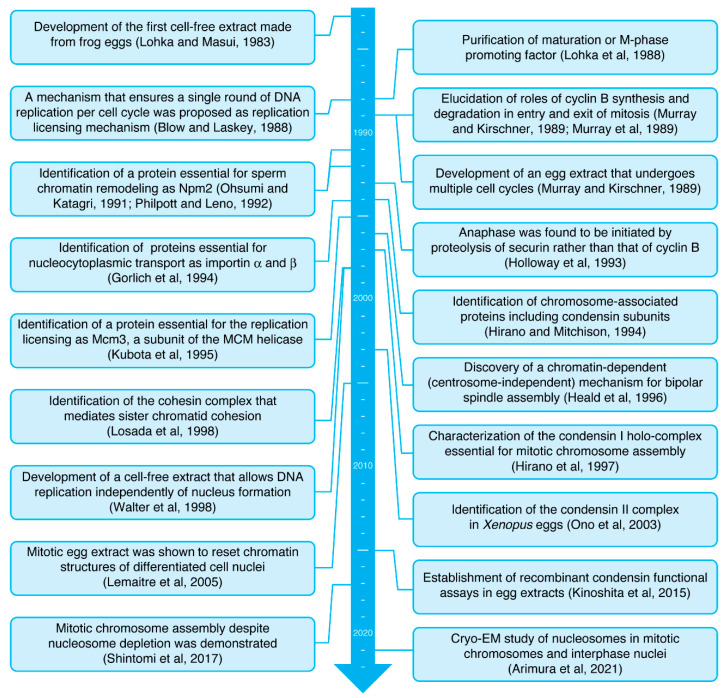
Timeline of studies using frog egg extracts [1,2,4,11,12,13,14,15,16,17,18,19,20,21,22,23,24,25,26,27].

Cell-free extracts made from frog eggs were first described for studying the large-scale transformations of chromatin structures by Yoshio Masui in 1983 [4]. This pioneering work demonstrated that the cytoplasmic extract was readily prepared from eggs of the northern leopard frog *Rana pipiens* by centrifugation (Figure 1A) and that interphase nuclei and mitotic chromosomes can be assembled in a test tube, by incubating sperm nuclei of the African clawed frog *Xenopus laevis* in the *Rana* egg extract (Figure 1B). A year later, a similar cell-free system using egg extracts and sperm nuclei of the toad *Bufo japonicus* was also reported [5]. Subsequent works have developed protocols for the extract preparation from *Xenopus* eggs (instead of *Rana* or *Bufo* eggs) and for precise manipulation of the cell cycle stages in an extract [6,7,8,9]. Thus, frog egg extracts have broadened our understanding of the basic mechanism of cell cycle regulation. In parallel, the application of egg extracts has gradually been extended to biochemical studies on cell cycle-regulated chromosomal events such as DNA replication in S phase and chromosome assembly and segregation in mitosis. One of the most powerful protocols in this experimental system is the depletion of a target protein from egg extracts with its specific antibody (immunodepletion). This sharply addresses the requirement of a given protein for the corresponding cellular process. It is also possible to replace an endogenous protein with a recombinant protein (e.g., a protein with site-directed mutants) and investigate the functionality of the recombinant one in the extract [10]. Furthermore, proteins that mediate various biological processes can be purified from an egg extract by conventional column chromatography [11]. In this way, many scientists who have used frog egg extracts benefited from their unique biochemical tractability and produced many historically important results in cell biology [1,2,4,11,12,13,14,15,16,17,18,19,20,21,22,23,24,25,26,27] (Figure 2).

## 3. Sperm Chromatin Remodeling: An Essential Preparatory Step for Chromosome Assembly in In Vitro Assays

In retrospect, frog egg extracts were originally developed with an aim of recapitulating the dynamic process of male pronucleus formation that naturally occurs in fertilized eggs, rather than an aim of studying the cell cycle [4,5]. It is no exaggeration to say that the current in vitro assays for mitotic chromosome research originated from these founding studies. In this section, the molecular mechanisms behind the early stage of male pronucleus formation and how they have been elucidated using cell-free assays are introduced.

**Figure 3:**
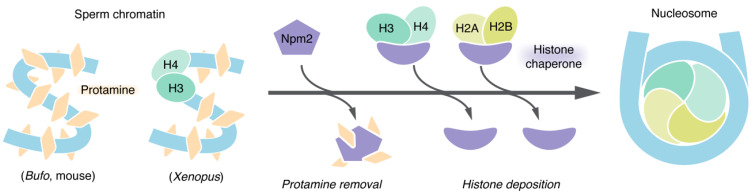
Sperm chromatin remodeling mediated by histone chaperones. Protamine occupies a large part of the proteinaceous components of sperm chromatin. Immediately after fertilization, protamine is removed from the sperm DNA by the histone chaperone Npm2 in the egg cytoplasm. Core histones instead are deposited on the DNA by actions of histone chaperones (e.g., Npm2, Nap1, NASP (also known as N1), HIRA, and Asf1), consequently promoting nucleosome assembly. In *Xenopus* (unlike *Bufo* or mouse), sperm nuclei contain adequate amounts of histones H3–H4, which later participate in nucleosome assembly in fertilized eggs.

Sperm is the male gamete that is specialized to transmit the paternal genome to the next generation through sexual reproduction. To smoothly accomplish this task, a sperm nucleus has a highly compact shape in most animals. The bulk of chromosomal DNA in mature sperm interacts with arginine-rich small polypeptides collectively known as protamines (also known as sperm-specific basic proteins in some amphibian species). Protamines replace nucleosomal histones during spermatogenesis, maintaining a highly compact state of the nuclei. However, such a non-canonical chromatin structure must be broken shortly after fertilization so that the paternal genome becomes competent for DNA replication and transcription. When sperm is incorporated into the egg cytoplasm, its nuclear envelope rapidly disappears and the chromatin swells. Protamines are then replaced with histones to assemble nucleosomes along the entire length of DNA [28,29] (Figure 3).

A series of these events, hereafter referred to as “sperm chromatin remodeling”, had been studied mainly by descriptive approaches such as light and electron microscopy until the early 1980s [29]. However, this situation drastically changed after the establishment of frog egg extracts by which the whole process of sperm chromatin remodeling can be recapitulated in a test tube. An outline of the cell-free assay is as follows. First, sperm nuclei isolated from testes of male frogs are treated with natural lecithins or non-ionic detergents to permeabilize their plasma and nuclear membranes. Next, the resultant “demenbranated” sperm nuclei are incubated in an egg extract at a temperature of around 20 °C. Consequently, sperm chromatin remodeling is completed within several minutes [4,5,15,30]. This indicates that activities sufficient for remodeling reside in egg extract. A protein responsible for both chromatin swelling and protamine removal was first identified as nucleoplasmin (currently referred to as Npm2) via fractionation of a *Bufo* egg extract [31]. A requirement of Npm2 for these events was confirmed by its immunodepletion from *Xenopus* egg extracts [30] (Figure 3). 

It is believed that nucleosome assembly on sperm-derived paternal DNA must be coupled with protamine removal to keep the DNA from being physically vulnerable and to facilitate pronucleus formation [32,33,34]. Biochemical reconstitution using purified factors provided a clue to the question of which protein is central for nucleosome assembly on paternal DNA. Npm2 in *X. laevis* was characterized as the first member of histone chaperones (a class of proteins that facilitate nucleosome assembly [35,36]), and its ability to properly deposit histones on naked plasmid DNA was intensively studied [37,38]. Therefore, it was a natural trend to test whether Npm2 fulfills a similar task on sperm-derived paternal DNA. As expected, by incubating demembranated sperm nuclei with purified Npm2 and core histones, not only protamine removal but also nucleosome assembly can be recapitulated [15,16]. Furthermore, a different experimental setup showed that Nap1 cooperates with Npm2 to efficiently promote nucleosome assembly on *Xenopus* sperm DNA [39]. Large quantities of various histone chaperones are known to exist in frog eggs [39,40,41,42,43,44]. It is therefore possible that several histone chaperones cooperatively facilitate nucleosome assembly on sperm DNA (Figure 3). 

## 4. Mitotic Chromosome Assembly Recapitulated in Egg Extracts

In the original cell-free assays using an “interphase” extract, sperm chromatin remodeling is followed by assembly of the nucleus, and then, a single round of DNA replication occurs in the nucleus [4]. After completion of DNA replication, mitosis can be induced by adding cyclin B, a protein required for activation of the mitotic kinase Cdk1 [45]. As a result, the nuclear envelope breaks down, and “duplicated chromosomes”, each of which is composed of a pair of sister chromatids, are assembled (Figure 1B) [4,45]. Conversely, a “mitotic” extract can be prepared from unfertilized eggs by soaking them in a buffer containing the Ca^2+^-chelating agent EGTA before centrifugation [6]. This is because the cell cycle of unfertilized frog eggs is arrested in metaphase of meiosis II by the activity known as a cytostatic factor, which is inactivated by a transient increase in the cytoplasmic Ca^2+^ ion upon fertilization [46,47]. When sperm nuclei are incubated in a mitotic extract, they undergo chromatin remodeling and are directly transformed into a cluster of chromosome-like structures, which consist of “single chromatids” [48] (Figure 1C). Although the former assay reproduces cellular events in a physiological order, the latter assay is more frequently used for chromosome studies because of its biochemical tractability.

Egg extracts can be further clarified by high-speed centrifugation at ~150,000× *g* (Figure 1A). The high-speed supernatant (HSS) of a mitotic extract retains the ability to recapitulate single chromatid assembly, whereas an interphase HSS fails to support nuclear assembly or DNA replication [7,48]. The use of the mitotic HSS allowed us to cleanly purify large quantities of chromosomes by single-step centrifugation. Taking advantage of this, major proteinaceous components of mitotic chromatids, collectively referred to as *Xenopus* chromosome-associated polypeptides (XCAPs), were isolated. The composition of XCAPs turned out to be simple, being composed of the subunits of the complex currently known as condensin I, topoisomerase IIα (topo IIα), and core and linker histones [1,2]. 

## 5. Mitotic Chromatids Can Be Made by Using Purified Proteins

The abovementioned simple composition of XCAPs prompted us to reason that it might be possible to produce structures similar to mitotic chromatids using purified proteins instead of egg extracts. In the beginning, this idea itself seemed extremely challenging because it remained to be determined how many regulatory proteins (i.e., proteins other than XCAPs) are involved in this process. However, through biochemical fractionation of M-HSS, we were able to narrow down the number of proteins necessary and sufficient for the reconstitution. It was finally demonstrated that structure resembling mitotic single chromatids can be reconstituted from only six purified proteins (condensin I, topo IIα, core histones, and three histone chaperones (Npm2, Nap1, and FACT)) and *Xenopus* sperm nuclei [3]. To be more precise, only core histones H2A-H2B are needed to be supplied because an adequate amount of H3-H4 retains in *Xenopus* sperm nuclei (Figure 3). Only three of these proteins, core histone, topo IIα, and condensin I, localize on the resultant structures, while the rest are histone chaperones that leave them after transient actions. The omission of either one of the three chaperones caused distinct defects in chromatin morphogenesis, indicating that they execute non-overlapping essential functions. Overall, the elementary process of chromosome assembly, which was previously thought to be extremely complex, is supported by a limited number of proteins. 

**Figure 4:**
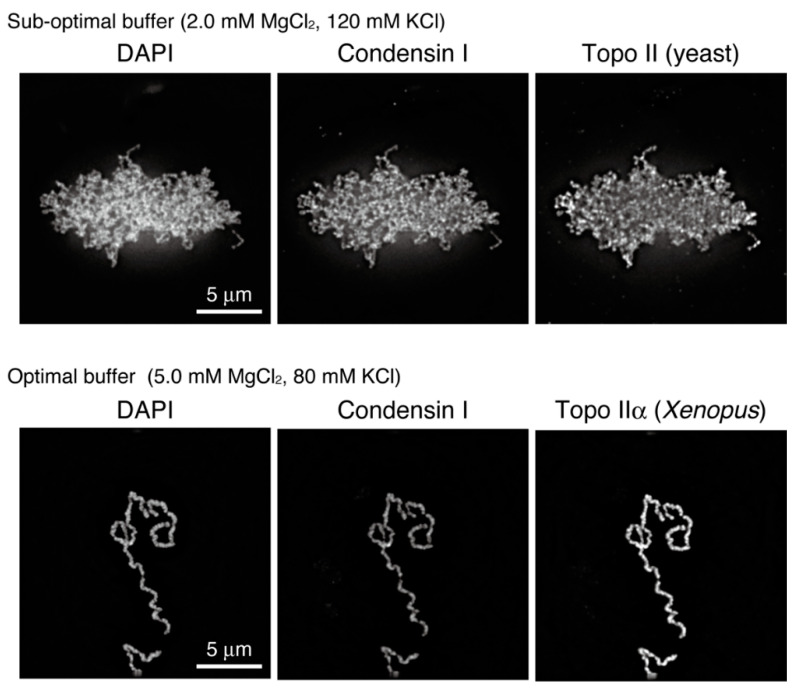
Mitotic chromatids reconstituted with purified factors. The final structures reconstituted under different conditions were analyzed by immunofluorescence. Note that preparations of topo II were also different between the two tested conditions: budding yeast topo II and *Xenopus laevis* topo IIα were used under sub-optimal and the optimal conditions, respectively.

However, the final structures reconstituted in the original assay, which were composed of mutually entangled chromatin fibers, were not necessarily identical to single chromatids assembled in cell-free assays using egg extracts (Figure 4, upper). To fill this gap, we first developed a protocol to evaluate the reconstituted structures using morphometric parameters and then surveyed the conditions in which the final structures become thicker and entanglements between them are mostly eliminated. As a successful outcome of these challenges, optimal buffer conditions for chromatid reconstitution were reported recently [49]. The structures reconstituted in this “second-generation” assay are morphologically indistinguishable from chromatids observed in the cell-free assay (Figure 4, lower). The systematic survey of buffer conditions revealed that functions of topo IIα are sensitive to salt concentrations. It has long been known that monovalent and divalent cations (e.g., K^+^ and Mg^2+^ ions) are enriched on mitotic chromosomes in vivo [50,51,52,53]. The reconstitution assays, in which ion atmospheres around chromatin can be manipulated, will be instrumental in addressing the physiological significance of chromosome-bound cations.

## 6. Our Current Understanding of Major Chromosome-Associated Proteins 

In the following subsections, to what extent the functions of the major proteinaceous components of mitotic chromatids and their regulation have been understood using cell-free extracts and reconstitution assays are outlined. 

### 6.1. Condensins: ATP-Utilizing Machines That Fold a DNA Strand into a Chromosome 

Condensin I is composed of two core subunits with ATP-binding domains (CAP-C/Smc4 and CAP-E/Smc2) and three regulatory subunits (CAP-D2, CAP-G, and CAP-H). Many eukaryotes have condensin II, which shares a common set of core subunits but has a different set of regulatory subunits (CAP-D3, CAP-G2, and CAP-H2) [54,55,56]. When sperm nuclei were incubated in an extract immunodepleted of both condensins, a cloud-like amorphous chromatin mass was produced, drawing the solid conclusion that condensins are indispensable for mitotic chromosome assembly [2,23,57]. Furthermore, cell-free assays using extracts depleted of either condensin I or II revealed that they have non-overlapping functions [23,58]. A series of analyses involving manipulation of the ratio of condensin I to II underscored that condensin I promotes lateral compaction perpendicular to the chromosome axis while condensin II contributes to longitudinal compaction along the axis [58]. Note that supporting results have been reported in chicken DT40 cells [59]. 

The recombinant complexes of condensins I and II expressed in insect cells have been demonstrated to rescue the defect in chromosome assembly caused by condensin depletion in the egg extract [25,60,61]. This protocol paves the way for addressing functionalities of mutant condensins with amino acid substitutions and those of “sub-complexes” in which either one(s) of the five subunits are lost. Recent analyses using a variety of recombinant complexes have revealed hitherto unappreciated functional crosstalk between the subunits and differences in regulatory mechanisms between condensins I and II [25,61,62,63]. 

**Figure 5:**
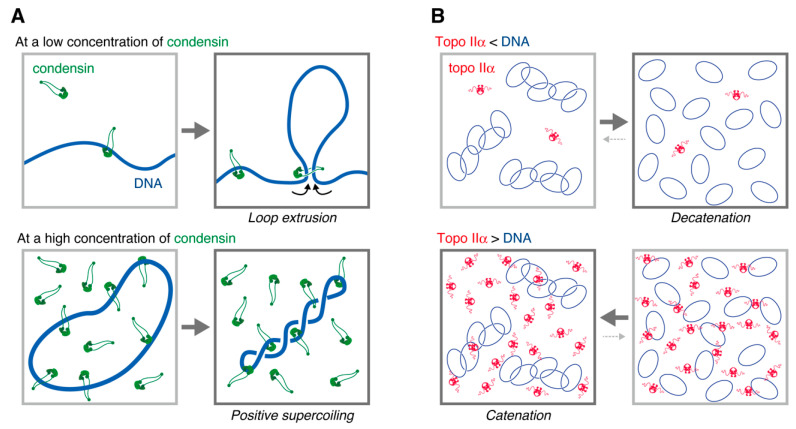
Condensin and topo IIα differently act on DNA depending on ambient environments. (**A**) Condensin binds a stretch of DNA and extrudes it into a loop. This activity is known to be detectable in a reaction mixture containing a low concentration of condensin (~1.0 nM) (upper panel). In contrast, when an excess amount of condensin I is mixed with relaxed circular DNA in the presence of topoisomerase I (which removes only compensatory negative supercoils), positive supercoils are created on the DNA (lower panel). (**B**) Topo IIα can catalyze both decatenation and catenation of circular DNAs. The equilibrium of these opposite reactions was tilted depending on the ratio of DNA to topo IIα.

*Xenopus* egg extracts have also led to important findings on regulations of condensin-mediated mechanochemical reactions. Multiple amino acid residues of condensin subunits are known to be phosphorylated during mitosis in *Xenopus* as well as in humans and budding yeast [2,64,65,66]. It is therefore possible to purify hyper-phosphorylated and hypo-phosphorylated forms of condensins using mitotic and interphase egg extracts, respectively. Only a mitotic form of condensin I can introduce positive supercoils into DNA in an ATP hydrolysis-dependent manner [67,68] (Figure 5A). The same is also true for condensins purified from human cells and budding yeast [65,66]. Furthermore, chromatid reconstitution using purified factors requires the mitotic phosphorylated form of condensin I [3]. It is thus believed that the positive supercoil introducing activity of condensin I is physiologically relevant. Conversely, increasing lines of evidence show that condensins are capable of extruding DNA loops in an ATP hydrolysis-dependent manner [63,69,70,71,72,73] (Figure 5A). Loop extrusion by condensins has been observed not only in an experimental setup made of purified proteins but also in a mitotic egg extract [74]. During the past years, several molecular mechanisms have been proposed for condensin-mediated loop extrusion (see reviews [75,76,77,78]). How positive supercoils and loops on a long stretch of genomic DNA, which are created by condensins, result in highly organized structures of mitotic chromosomes is one of the biggest open questions.

### 6.2. Topo IIα: A Catalyst for Disentanglement and Entanglement of Chromosomal DNA

Topo IIα is an enzyme that manipulates DNA tangles and supercoils through a unique mechanism involving cleavage and re-ligation of a double-stranded DNA (known as the strand passage reaction) [79,80]. It is therefore believed that topo IIα facilitates an essential initial step for chromosome assembly by resolving inter-chromosomal entanglements. In the single chromatid assembly assay using *Xenopus* egg extracts, depletion of topo IIα completely blocks chromatid individualization, leaving a banana-shaped chromatin mass derived from the *Xenopus* sperm nucleus [48]. An egg extract cell-free assay using erythrocyte nuclei as substrates [81] has also shown the requirement of topo IIα for chromatid assembly [82]. This excludes the possibility that the requirement of topo IIα is limited to sperm nucleus substrates, which are protamine-based “non-canonical” chromatin.

Our recent results, which were obtained from a combination of chromatid reconstitution assays and enzymological assays, strongly suggested that topo IIα first resolves inter-chromatid entanglements to drive chromatid individualization and then generates intra-chromatid entanglements to promote chromatid thickening [49]. Only the latter process requires the C-terminal domain (CTD) of topo IIα, which is known to be required for its chromosomal binding [83]. Consistently, topo IIα catalyzes different reactions on circular DNA (i.e., decatenation and catenation) depending on molecular crowding in the ambient environment (Figure 5B). Analogous to this, condensin I also differently acts depending on its concentration: it creates DNA loops at a relatively low concentration [69], whereas positive supercoiling requires a considerably high concentration of condensin I [67] (Figure 5A). In the future, it will be important to understand how DNA and chromatin structures are transformed by the cooperative actions of topo IIα and condensins in the crowded environment that were naturally created around the axis of each chromatid.

### 6.3. Histones: Dynamic Bricks of Chromosomes

Although the core histones occupy the largest parts of the whole protein mass of mitotic chromosomes [1,84,85], to what extent they might directly contribute to large-scale chromatid assembly had not been studied until recently. The chromatid reconstitution assay allowed us to address this question from a fresh angle. At present, successful reconstitution relies on the specific combination of core histone H2A-H2B, namely, that of N-terminally truncated versions of H2A.X-F (an embryo-specific variant of H2A) and canonical H2B [3,86]. This result has two interesting implications. First, the use of histones lacking the N-terminal tails might bypass potential requirements for post-translational modifications in these regions, thus minimizing the number of factors required for reconstitution. Second, the C-terminus of H2A.X-F is extended and acidic, unlike that of canonical H2A. Such unique characteristics might modulate histone–DNA interaction, ensuring the productive actions of topo IIα and condensin I on chromatin. In addition, the reconstitution assay also uncovered the otherwise cryptic importance of the histone chaperone FACT, which has been implicated in the destabilization of nucleosomes [87]. Taken all together, it is most likely that the dynamic nature of nucleosomes underlies large-scale chromatid assembly. Notably, a recent paper using the cell-free assays reported that the general transcription factor TFIIH alters nucleosome structures to generate a chromatin environment for productive actions of condensins in mitosis [88].

Is nucleosome assembly per se an essential prerequisite for chromatid assembly? This naïve question was addressed by modifying the single chromatid assembly assay using the *Xenopus* egg extract. Briefly, mouse sperm nuclei, which barely contain all core histones, unlike *Xenopus* sperm nuclei [89] (Figure 3), were used as substrates. It was first confirmed that this heterologous cell-free system supports nucleosome assembly on mouse sperm DNAs and converts them into a cluster of rod-shaped single chromatids [26]. This system also allowed us to impede the whole process of nucleosome assembly on sperm DNA by depleting the histone chaperone Asf1 from an egg extract [90]. In the Asf1-depleted extract, mitotic chromatid-like structures could be assembled despite the absence of nucleosomes. The resultant “nucleosome-depleted” chromatid was composed of the condensin-enriched central axis and hazy chromatin masses surrounding it. It is most likely that nucleosomes themselves do not play a vital role in folding a centimeters-long genomic DNA into micrometers-long chromatids, although they contribute to the compaction of DNA loops emanated from the chromatid axes.

It was formerly thought that linker histones have indispensable roles in mitotic chromosome assembly because they are known to be heavily phosphorylated by the mitotic kinase cyclin B-Cdk1 [91,92,93]. The cell-free assay of egg extracts allowed for more direct investigation of the linker histones’ roles. The embryonic variant H1.8 (previously termed B4, H1M, or H1X) is the unique linker histone that resides in *Xenopus* eggs [31,94], whose binding to the nucleosome dyad axis in mitotic chromosomes was demonstrated in a recent cryo-EM analysis [27]. Nevertheless, depletion of H1.8 from the mitotic egg extracts caused no discernible defects in the morphology of single chromatids assembled in the cell-free assay [95]. It was later demonstrated that single chromatids recruit reduced levels of H1.8, prompting re-investigation of its role in more physiological chromosomes that have undergone DNA replication. Duplicated chromosomes assembled in an H1.8-depleted egg extract are longitudinally elongated and contain greater amounts of condensins and topo IIα than those assembled in a mock-depleted control extract [96,97]. Related to this, chromatin loading of condensins and topo IIα increases on nucleosome-depleted DNA in egg extracts [26,34]. In summary, currently available data for manipulation of core and linker histones in the cell-free and reconstitution assays strongly suggest that nucleosome dynamics underlie proper actions of condensins and topo IIα.

## 7. Conclusions

During the past 40 years since the establishment of frog egg extracts, our understanding of large-scale chromatin transformations including sperm chromatin remodeling and mitotic chromosome assembly has been greatly strengthened. Now that it is possible to reconstitute chromosome-like structures with purified factors, we are moving into a new era to solve how major chromosomal proteins cooperatively act. One of the most important questions is how condensins and topo IIα act on nucleosome templates. The use of mouse sperm nuclei as starting materials in the reconstitution assay will pave the way for the introduction of various mutant forms of histones as well as those of condensins and topo IIα. The long-term goal in this research field is to comprehensively understand how a chromatin fiber is folded into a mitotic chromatid. To this end, we must deeply investigate in vitro assembled chromosomes described here by using recently emerging analytical technologies, including chromosome-conformation capture analysis (Hi-C) [97,98,99,100,101,102] and mechanical manipulation of chromosomes [103,104,105,106,107].

In addition, I wonder if the chromatid reconstitution assay might be of great use for practical research. For instance, it is known that, for successful animal cloning, a donor nucleus isolated from somatic cells must be injected into a “mitotic” recipient enucleated egg [108,109]. In relation to this animal cloning protocol, when nuclei isolated from terminally differentiated cells undergo chromosome assembly in a mitotic egg extract, they become competent for DNA replication in subsequent interphase [24]. It might be possible to improve the success rate of cloning by pretreating donor nuclei with defined purified factors that participate in mitotic chromatid assembly. Thus, the in vitro assays described here will broaden our horizons in chromatin biology and neighboring fields.

## References

[1] Hirano T., Mitchison T.J. (1994). A heterodimeric coiled-coil protein required for mitotic chromosome condensation in vitro. Cell.

[2] Hirano T., Kobayashi R., Hirano M. (1997). Condensins, chromosome condensation protein complexes containing XCAP-C, XCAP-E and a *Xenopus* homolog of the Drosophila Barren protein. Cell.

[3] Shintomi K., Takahashi T.S., Hirano T. (2015). Reconstitution of mitotic chromatids with a minimum set of purified factors. Nat. Cell Biol..

[4] Lohka M.J., Masui Y. (1983). Formation in vitro of sperm pronuclei and mitotic chromosomes induced by amphibian ooplasmic components. Science.

[5] Iwao Y., Katagiri C. (1984). In vitro induction of sperm nucleus decondensation by cytosol from mature toad eggs. J. Exp. Zool..

[6] Lohka M.J., Masui Y. (1984). Effects of Ca^2+^ ions on the formation of metaphase chromosomes and sperm pronuclei in cell-free preparations from unactivated Rana pipiens eggs. Dev. Biol..

[7] Lohka M.J., Maller J.L. (1985). Induction of nuclear envelope breakdown, chromosome condensation, and spindle formation in cell-free extracts. J. Cell Biol..

[8] Miake-Lye R., Kirschner M.W. (1985). Induction of early mitotic events in a cell-free system. Cell.

[9] Murray A.W. (1991). Cell cycle extracts. Methods Cell Biol..

[10] Jenness C., Wynne D.J., Funabiki H. (2018). Protein Immunodepletion and Complementation in *Xenopus laevis* Egg Extracts. Cold Spring Harb. Protoc..

[11] Lohka M.J., Hayes M.K., Maller J.L. (1988). Purification of maturation-promoting factor, an intracellular regulator of early mitotic events. Proc. Natl. Acad. Sci. USA.

[12] Blow J.J., Laskey R.A. (1988). A role for the nuclear envelope in controlling DNA replication within the cell cycle. Nature.

[13] Murray A.W., Kirschner M.W. (1989). Cyclin synthesis drives the early embryonic cell cycle. Nature.

[14] Murray A.W., Solomon M.J., Kirschner M.W. (1989). The role of cyclin synthesis and degradation in the control of maturation promoting factor activity. Nature.

[15] Ohsumi K., Katagiri C. (1991). Characterization of the ooplasmic factor inducing decondensation of and protamine removal from toad sperm nuclei: Involvement of nucleoplasmin. Dev. Biol..

[16] Philpott A., Leno G.H. (1992). Nucleoplasmin remodels sperm chromatin in *Xenopus* egg extracts. Cell.

[17] Holloway S.L., Glotzer M., King R.W., Murray A.W. (1993). Anaphase is initiated by proteolysis rather than by the inactivation of maturation-promoting factor. Cell.

[18] Görlich D., Prehn S., Laskey R.A., Hartmann E. (1994). Isolation of a protein that is essential for the first step of nuclear protein import. Cell.

[19] Kubota Y., Mimura S., Nishimoto S., Takisawa H., Nojima H. (1995). Identification of the yeast MCM3-related protein as a component of *Xenopus* DNA replication licensing factor. Cell.

[20] Heald R., Tournebize R., Blank T., Sandaltzopoulos R., Becker P., Hyman A., Karsenti E. (1996). Self-organization of microtubules into bipolar spindles around artificial chromosomes in *Xenopus* egg extracts. Nature.

[21] Losada A., Hirano M., Hirano T. (1998). Identification of *Xenopus* SMC protein complexes required for sister chromatid cohesion. Genes Dev..

[22] Walter J., Sun L., Newport J. (1998). Regulated chromosomal DNA replication in the absence of a nucleus. Mol. Cell.

[23] Ono T., Losada A., Hirano M., Myers M.P., Neuwald A.F., Hirano T. (2003). Differential contributions of condensin I and condensin II to mitotic chromosome architecture in vertebrate cells. Cell.

[24] Lemaitre J.M., Danis E., Pasero P., Vassetzky Y., Méchali M. (2005). Mitotic remodeling of the replicon and chromosome structure. Cell.

[25] Kinoshita K., Kobayashi T.J., Hirano T. (2015). Balancing acts of two HEAT subunits of condensin I support dynamic assembly of chromosome axes. Dev. Cell.

[26] Shintomi K., Inoue F., Watanabe H., Ohsumi K., Ohsugi M., Hirano T. (2017). Mitotic chromosome assembly despite nucleosome depletion in *Xenopus* egg extracts. Science.

[27] Arimura Y., Shih R.M., Froom R., Funabiki H. (2021). Structural features of nucleosomes in interphase and metaphase chromosomes. Mol. Cell.

[28] Kunkle M., Longo F.J., Magun B.E. (1978). Nuclear protein changes in the maternally and paternally derived chromatin at fertilization. J. Exp. Zool..

[29] Longo F.J. (1985). Pronuclear events during fertilization. Biology of Fertilization.

[30] Philpott A., Leno G.H., Laskey R.A. (1991). Sperm decondensation in *Xenopus* egg cytoplasm is mediated by nucleoplasmin. Cell.

[31] Ohsumi K., Katagiri C. (1991). Occurrence of H1 subtypes specific to pronuclei and cleavage-stage cell nuclei of anuran amphibians. Dev. Biol..

[32] Loppin B., Bonnefoy E., Anselme C., Laurencon A., Karr T.L., Couble P. (2005). The histone H3.3 chaperone HIRA is essential for chromatin assembly in the male pronucleus. Nature.

[33] Inoue A., Zhang Y. (2014). Nucleosome assembly is required for nuclear pore complex assembly in mouse zygotes. Nat. Struct. Mol. Biol..

[34] Zierhut C., Jenness C., Kimura H., Funabiki H. (2014). Nucleosomal regulation of chromatin composition and nuclear assembly revealed by histone depletion. Nat. Struct. Mol. Biol..

[35] Onikubo T., Shechter D. (2016). Chaperone-mediated chromatin assembly and transcriptional regulation in *Xenopus laevis*. Int. J. Dev. Biol..

[36] Hammond C.M., Strømme C.B., Huang H., Patel D.J., Groth A. (2017). Histone chaperone networks shaping chromatin function. Nat. Rev. Mol. Cell Biol..

[37] Laskey R.A., Honda B.M., Mills A.D., Finch J.T. (1978). Nucleosomes are assembled by an acidic protein which binds histones and transfers them to DNA. Nature.

[38] Earnshaw W.C., Honda B.M., Laskey R.A., Thomas J.O. (1980). Assembly of nucleosomes: The reaction involving X. laevis nucleoplasmin. Cell.

[39] Shintomi K., Iwabuchi M., Saeki H., Ura K., Kishimoto T., Ohsumi K. (2005). Nucleosome assembly protein-1 is a linker histone chaperone in *Xenopus* eggs. Proc. Natl. Acad. Sci. USA.

[40] Kleinschmidt J.A., Dingwall C., Maier G., Franke W.W. (1986). Molecular characterization of a karyophilic, histone-binding protein: cDNA cloning, amino acid sequence and expression of nuclear protein N1/N2 of *Xenopus laevis*. EMBO J..

[41] Dilworth S.M., Black S.J., Laskey R.A. (1987). Two complexes that contain histones are required for nucleosome assembly in vitro: Role of nucleoplasmin and N1 in *Xenopus* egg extracts. Cell.

[42] Quivy J.P., Grandi P., Almouzni G. (2001). Dimerization of the largest subunit of chromatin assembly factor 1: Importance in vitro and during *Xenopus* early development. EMBO J..

[43] Ray-Gallet D., Quivy J.P., Scamps C., Martini E.M., Lipinski M., Almouzni G. (2002). HIRA is critical for a nucleosome assembly pathway independent of DNA synthesis. Mol. Cell.

[44] Wuhr M., Freeman R.M., Presler M., Horb M.E., Peshkin L., Gygi S.P., Kirschner M.W. (2014). Deep proteomics of the *Xenopus laevis* egg using an mRNA-derived reference database. Curr. Biol..

[45] Shintomi K., Hirano T. (2017). A Sister Chromatid Cohesion Assay Using *Xenopus* Egg Extracts. Methods Mol. Biol..

[46] Masui Y. (1974). A cytostatic factor in amphibian oocytes: Its extraction and partial characterization. J. Exp. Zool..

[47] Liu J., Grimison B., Maller J.L. (2007). New insight into metaphase arrest by cytostatic factor: From establishment to release. Oncogene.

[48] Hirano T., Mitchison T.J. (1993). Topoisomerase II does not play a scaffolding role in the organization of mitotic chromosomes assembled in *Xenopus* egg extracts. J. Cell Biol..

[49] Shintomi K., Hirano T. (2021). Guiding functions of the C-terminal domain of topoisomerase IIalpha advance mitotic chromosome assembly. Nat. Commun..

[50] Lewis C.D., Laemmli U.K. (1982). Higher order metaphase chromosome structure: Evidence for metalloprotein interactions. Cell.

[51] Strick R., Strissel P.L., Gavrilov K., Levi-Setti R. (2001). Cation-chromatin binding as shown by ion microscopy is essential for the structural integrity of chromosomes. J. Cell Biol..

[52] Phengchat R., Takata H., Morii K., Inada N., Murakoshi H., Uchiyama S., Fukui K. (2016). Calcium ions function as a booster of chromosome condensation. Sci. Rep..

[53] Maeshima K., Matsuda T., Shindo Y., Imamura H., Tamura S., Imai R., Kawakami S., Nagashima R., Soga T., Noji H. (2018). A Transient Rise in Free Mg(2+) Ions Released from ATP-Mg Hydrolysis Contributes to Mitotic Chromosome Condensation. Curr. Biol..

[54] Hirano T. (2016). Condensin-Based Chromosome Organization from Bacteria to Vertebrates. Cell.

[55] Uhlmann F. (2016). SMC complexes: From DNA to chromosomes. Nat. Rev. Mol. Cell Biol..

[56] Hassler M., Shaltiel I.A., Haering C.H. (2018). Towards a Unified Model of SMC Complex Function. Curr. Biol..

[57] Wignall S.M., Deehan R., Maresca T.J., Heald R. (2003). The condensin complex is required for proper spindle assembly and chromosome segregation in *Xenopus* egg extracts. J. Cell Biol..

[58] Shintomi K., Hirano T. (2011). The relative ratio of condensin I to II determines chromosome shapes. Genes Dev..

[59] Green L.C., Kalitsis P., Chang T.M., Cipetic M., Kim J.H., Marshall O., Turnbull L., Whitchurch C.B., Vagnarelli P., Samejima K. (2012). Contrasting roles of condensin I and condensin II in mitotic chromosome formation. J. Cell Sci..

[60] Onn I., Aono N., Hirano M., Hirano T. (2007). Reconstitution and subunit geometry of human condensin complexes. EMBO J..

[61] Yoshida M.M., Kinoshita K., Aizawa Y., Tane S., Yamashita D., Shintomi K., Hirano T. (2022). Molecular dissection of condensin II-mediated chromosome assembly using in vitro assays. bioRxiv.

[62] Hara K., Kinoshita K., Migita T., Murakami K., Shimizu K., Takeuchi K., Hirano T., Hashimoto H. (2019). Structural basis of HEAT-kleisin interactions in the human condensin I subcomplex. EMBO Rep..

[63] Kinoshita K., Tsubota Y., Tane S., Aizawa Y., Sakata R., Takeuchi K., Shintomi K., Nishiyama T., Hirano T. (2022). A loop extrusion-independent mechanism contributes to condensin I-mediated chromosome shaping. J. Cell Biol..

[64] Kimura K., Cuvier O., Hirano T. (2001). Chromosome condensation by a human condensin complex in *Xenopus* egg extracts. J. Biol. Chem..

[65] Takemoto A., Kimura K., Yokoyama S., Hanaoka F. (2004). Cell cycle-dependent phosphorylation, nuclear localization, and activation of human condensin. J. Biol. Chem..

[66] St-Pierre J., Douziech M., Bazile F., Pascariu M., Bonneil E., Sauvé V., Ratsima H., D’Amours D. (2009). Polo kinase regulates mitotic chromosome condensation by hyperactivation of condensin DNA supercoiling activity. Mol. Cell.

[67] Kimura K., Hirano T. (1997). ATP-dependent positive supercoiling of DNA by 13S condensin: A biochemical implication for chromosome condensation. Cell.

[68] Kimura K., Hirano M., Kobayashi R., Hirano T. (1998). Phosphorylation and activation of 13S condensin by Cdc2 in vitro. Science.

[69] Ganji M., Shaltiel I.A., Bisht S., Kim E., Kalichava A., Haering C.H., Dekker C. (2018). Real-time imaging of DNA loop extrusion by condensin. Science.

[70] Kim E., Kerssemakers J., Shaltiel I.A., Haering C.H., Dekker C. (2020). DNA-loop extruding condensin complexes can traverse one another. Nature.

[71] Kong M., Cutts E.E., Pan D., Beuron F., Kaliyappan T., Xue C., Morris E.P., Musacchio A., Vannini A., Greene E.C. (2020). Human Condensin I and II Drive Extensive ATP-Dependent Compaction of Nucleosome-Bound DNA. Mol. Cell.

[72] Ryu J.K., Rah S.H., Janissen R., Kerssemakers J.W.J., Bonato A., Michieletto D., Dekker C. (2022). Condensin extrudes DNA loops in steps up to hundreds of base pairs that are generated by ATP binding events. Nucleic Acids Res..

[73] Shaltiel I.A., Datta S., Lecomte L., Hassler M., Kschonsak M., Bravo S., Stober C., Ormanns J., Eustermann S., Haering C.H. (2022). A hold-and-feed mechanism drives directional DNA loop extrusion by condensin. Science.

[74] Golfier S., Quail T., Kimura H., Brugués J. (2020). Cohesin and condensin extrude DNA loops in a cell cycle-dependent manner. eLife.

[75] Yatskevich S., Rhodes J., Nasmyth K. (2019). Organization of Chromosomal DNA by SMC Complexes. Annu. Rev. Genet..

[76] Davidson I.F., Peters J.M. (2021). Genome folding through loop extrusion by SMC complexes. Nat. Rev. Mol. Cell Biol..

[77] Higashi T.L., Uhlmann F. (2022). SMC complexes: Lifting the lid on loop extrusion. Curr. Opin. Cell Biol..

[78] Oldenkamp R., Rowland B.D. (2022). A walk through the SMC cycle: From catching DNAs to shaping the genome. Mol. Cell.

[79] Wang J.C. (2002). Cellular roles of DNA topoisomerases: A molecular perspective. Nat. Rev. Mol. Cell Biol..

[80] Nitiss J.L. (2009). DNA topoisomerase II and its growing repertoire of biological functions. Nat. Rev. Cancer.

[81] Newport J., Spann T. (1987). Disassembly of the nucleus in mitotic extracts: Membrane vesicularization, lamin disassembly, and chromosome condensation are independent processes. Cell.

[82] Adachi Y., Luke M., Laemmli U.K. (1991). Chromosome assembly in vitro: Topoisomerase II is required for condensation. Cell.

[83] Linka R.M., Porter A.C., Volkov A., Mielke C., Boege F., Christensen M.O. (2007). C-terminal regions of topoisomerase IIalpha and IIbeta determine isoform-specific functioning of the enzymes in vivo. Nucleic Acids Res..

[84] Ohta S., Bukowski-Wills J.C., Sanchez-Pulido L., Alves Fde L., Wood L., Chen Z.A., Platani M., Fischer L., Hudson D.F., Ponting C.P. (2010). The protein composition of mitotic chromosomes determined using multiclassifier combinatorial proteomics. Cell.

[85] Booth D.G., Beckett A.J., Molina O., Samejima I., Masumoto H., Kouprina N., Larionov V., Prior I.A., Earnshaw W.C. (2016). 3D-CLEM Reveals that a Major Portion of Mitotic Chromosomes Is Not Chromatin. Mol. Cell.

[86] Shechter D., Chitta R.K., Xiao A., Shabanowitz J., Hunt D.F., Allis C.D. (2009). A distinct H2A.X isoform is enriched in *Xenopus laevis* eggs and early embryos and is phosphorylated in the absence of a checkpoint. Proc. Natl. Acad. Sci. USA.

[87] Zhou K., Liu Y., Luger K. (2020). Histone chaperone FACT FAcilitates Chromatin Transcription: Mechanistic and structural insights. Curr. Opin. Struct. Biol..

[88] Haase J., Chen R., Parker W.M., Bonner M.K., Jenkins L.M., Kelly A.E. (2022). The TFIIH complex is required to establish and maintain mitotic chromosome structure. eLife.

[89] Brykczynska U., Hisano M., Erkek S., Ramos L., Oakeley E.J., Roloff T.C., Beisel C., Schübeler D., Stadler M.B., Peters A.H. (2010). Repressive and active histone methylation mark distinct promoters in human and mouse spermatozoa. Nat. Struct. Mol. Biol..

[90] Ray-Gallet D., Quivy J.P., Sillje H.W., Nigg E.A., Almouzni G. (2007). The histone chaperone Asf1 is dispensable for direct de novo histone deposition in *Xenopus* egg extracts. Chromosoma.

[91] Bradbury E.M., Inglis R.J., Matthews H.R. (1974). Control of cell division by very lysine rich histone (F1) phosphorylation. Nature.

[92] Mueller R.D., Yasuda H., Bradbury E.M. (1985). Phosphorylation of histone H1 through the cell cycle of Physarum polycephalum. 24 sites of phosphorylation at metaphase. J. Biol. Chem..

[93] Langan T.A., Gautier J., Lohka M., Hollingsworth R., Moreno S., Nurse P., Maller J., Sclafani R.A. (1989). Mammalian growth-associated H1 histone kinase: A homolog of cdc2+/CDC28 protein kinases controlling mitotic entry in yeast and frog cells. Mol. Cell. Biol..

[94] Smith R.C., Dworkin-Rastl E., Dworkin M.B. (1988). Expression of a histone H1-like protein is restricted to early *Xenopus* development. Genes Dev..

[95] Ohsumi K., Katagiri C., Kishimoto T. (1993). Chromosome condensation in *Xenopus* mitotic extracts without histone H1. Science.

[96] Maresca T.J., Freedman B.S., Heald R. (2005). Histone H1 is essential for mitotic chromosome architecture and segregation in *Xenopus laevis* egg extracts. J. Cell Biol..

[97] Choppakatla P., Dekker B., Cutts E.E., Vannini A., Dekker J., Funabiki H. (2021). Linker histone H1.8 inhibits chromatin binding of condensins and DNA topoisomerase II to tune chromosome length and individualization. eLife.

[98] Naumova N., Imakaev M., Fudenberg G., Zhan Y., Lajoie B.R., Mirny L.A., Dekker J. (2013). Organization of the mitotic chromosome. Science.

[99] Kakui Y., Rabinowitz A., Barry D.J., Uhlmann F. (2017). Condensin-mediated remodeling of the mitotic chromatin landscape in fission yeast. Nat. Genet..

[100] Lazar-Stefanita L., Scolari V.F., Mercy G., Muller H., Guérin T.M., Thierry A., Mozziconacci J., Koszul R. (2017). Cohesins and condensins orchestrate the 4D dynamics of yeast chromosomes during the cell cycle. EMBO J..

[101] Schalbetter S.A., Goloborodko A., Fudenberg G., Belton J.M., Miles C., Yu M., Dekker J., Mirny L., Baxter J. (2017). SMC complexes differentially compact mitotic chromosomes according to genomic context. Nat. Cell Biol..

[102] Gibcus J.H., Samejima K., Goloborodko A., Samejima I., Naumova N., Nuebler J., Kanemaki M.T., Xie L., Paulson J.R., Earnshaw W.C. (2018). A pathway for mitotic chromosome formation. Science.

[103] Almagro S., Riveline D., Hirano T., Houchmandzadeh B., Dimitrov S. (2004). The mitotic chromosome is an assembly of rigid elastic axes organized by structural maintenance of chromosomes (SMC) proteins and surrounded by a soft chromatin envelope. J. Biol. Chem..

[104] Yan J., Maresca T.J., Skoko D., Adams C.D., Xiao B., Christensen M.O., Heald R., Marko J.F. (2007). Micromanipulation studies of chromatin fibers in *Xenopus* egg extracts reveal ATP-dependent chromatin assembly dynamics. Mol. Biol. Cell.

[105] Xiao B., Freedman B.S., Miller K.E., Heald R., Marko J.F. (2012). Histone H1 compacts DNA under force and during chromatin assembly. Mol. Biol. Cell.

[106] Sun M., Biggs R., Hornick J., Marko J.F. (2018). Condensin controls mitotic chromosome stiffness and stability without forming a structurally contiguous scaffold. Chromosome Res..

[107] Meijering A.E.C., Sarlós K., Nielsen C.F., Witt H., Harju J., Kerklingh E., Haasnoot G.H., Bizard A.H., Heller I., Broedersz C.P. (2022). Nonlinear mechanics of human mitotic chromosomes. Nature.

[108] Wakayama T., Perry A.C., Zuccotti M., Johnson K.R., Yanagimachi R. (1998). Full-term development of mice from enucleated oocytes injected with cumulus cell nuclei. Nature.

[109] Egli D., Rosains J., Birkhoff G., Eggan K. (2007). Developmental reprogramming after chromosome transfer into mitotic mouse zygotes. Nature.

